# Absence of Depressive and Anxious Behavior with Genetic Dysregulation in Adult C57Bl/6J Mice after Prenatal Exposure to Ionizing Radiation

**DOI:** 10.3390/ijms24108466

**Published:** 2023-05-09

**Authors:** Christine Lalonde, Shayenthiran Sreetharan, Alyssa Murray, Lisa Stoa, Mary Ellen Cybulski, Allison Kennedy, Nicholas Landry, Amy Stillar, Sandhya Khurana, Sujeenthar Tharmalingam, Joanna Wilson, Neelam Khaper, Simon J. Lees, Douglas Boreham, T. C. Tai

**Affiliations:** 1 Biomolecular Sciences, Laurentian University, Sudbury, ON P3E2C6, Canada; 2 School of Natural Sciences, Laurentian University, Sudbury, ON P3E2C6, Canada; 3 Medical Sciences Division, NOSM University, Sudbury, ON P3E2C6, Canada; 4 Department of Biology, McMaster University, Hamilton, ON L8S4L8, Canada; 5 Department of Psychology, Nipissing University, North Bay, ON P1B8L7, Canada

**Keywords:** radiation, fetal programming, late gestation, depression, anxiety

## Abstract

The exposure of ionizing radiation during early gestation often leads to deleterious and even lethal effects; however, few extensive studies have been conducted on late gestational exposures. This research examined the behavior al effects of C57Bl/6J mouse offspring exposed to low dose ionizing gamma irradiation during the equivalent third trimester. Pregnant dams were randomly assigned to sham or exposed groups to either low dose or sublethal dose radiation (50, 300, or 1000 mGy) at gestational day 15. Adult offspring underwent a behavioral and genetic analysis after being raised under normal murine housing conditions. Our results indicate very little change in the behavioral tasks measuring general anxiety, social anxiety, and stress-management in animals exposed prenatally across the low dose radiation conditions. Quantitative real-time polymerase chain reactions were conducted on the cerebral cortex, hippocampus, and cerebellum of each animal; results indicate some dysregulation in markers of DNA damage, synaptic activity, reactive oxygen species (ROS) regulation, and methylation pathways in the offspring. Together, our results provide evidence in the C57Bl/6J strain, that exposure to sublethal dose radiation (<1000 mGy) during the last period of gestation leads to no observable changes in behaviour when assessed as adults, although some changes in gene expression were observed for specific brain regions. These results indicate that the level of oxidative stress occurring during late gestation for this mouse strain is not sufficient for a change in the assessed behavioral phenotype, but results in some modest dysregulation of the genetic profile of the brain.

## 1. Introduction

Ionizing radiation, defined as sufficient incident energy to remove an electron from an atom, can create a cascade of physiological effects at the molecular level, leading to organ and system dysfunction or failure at sufficient dosage exposures [1]. Catastrophic incidents such as nuclear warhead detonation and nuclear power plant failures have propagated fear and avoidance of radiation through the workplace, medical interventions, and even of personal devices, regardless of mortality rates and statistics surrounding these exposures [2,3,4,5]. Dosimetry and exposure-rate research has provided evidence that early embryonic and fetal exposures can lead to termination, significant adverse birth defects, or low birth weights [1,6,7,8].

During the third trimester in humans, neural development is characterized by increased neuronal organization, migration, dendritogenesis, and synaptogenesis; all pertinent to healthy cognition and behavioral processes [9,10]. Medical diagnostic interventions and therapies that involve radiation during pregnancy are, arguably, of concern to the health and development of the fetus, even during the third trimester, which may not be critical for survival, but is highly sensitive and relevant for positive mental health outcomes [1,11,12]. Prenatal exposure in mice to x-ray doses up to 1000 mGy during early gestation has resulted in deficiencies in locomotor activity and spatial memory [13]. Forty-one genes related to p53 signaling, DNA damage, apoptosis, and cell signaling were differentially expressed. Further, x-ray exposures of 1000 mGy to C57Bl/6J mice on gestational day 11 altered post-synaptic density protein 95 (PSD95) in the hippocampi [14]. Swiss albino mice were exposed to low levels of gamma radiation between gestational days 11 and 19 and offspring were tested for a variety of behavioural responses, and it was reported that 500 mGy exposures produced activity, anxiety, and memory deficiencies in the 3-month-old offspring [15].

Fetal programming describes the impact of the in-utero environment on the phenotype of the offspring, whereby adverse environments lead to predispositions for adult diseases [16,17,18]. Deficient maternal diets, chronic exposures of stress hormones (glucocorticoids), and exposures to low doses of ionizing radiation have been linked to adulthood metabolic disorders which are associated with low birthweight, changes in blood-pressure, and abnormal adult behavioral function [1,19,20,21,22,23]. Fetal programming may occur through multiple mechanisms. First, activation of stress hormones may alter methylation of the genome; when glucocorticoids (GC) are circulating in high levels and overcome the placental enzymatic barrier, they bind to glucocorticoid response elements (GRE) in GC receptors, allowing changes in the methylation of CpG islands [24,25]. Depending on the location of methylation, the number of methyl groups, and the nucleic acid methylated, a gene may be silenced or activated, affecting physiologic processes that may adversely affect the metabolic and behavioural phenotype of the offspring [26,27]. Arguably, this process may be described as an adaptive response to a deficient uterine environment; low prenatal nutrition, for example, predicts a low-caloric diet for the offspring. A “thrifty phenotype” would be considered an adaptive response to a low-nutrient environment; conversely, when faced with a nutrient-rich environment, the response is aversive, leading to the development of adiposity, hypertension, and diabetes [28,29,30,31]. Second, stressors that induce DNA damage and increase levels of reactive oxygen species (ROS) stimulate a series of cellular responses, such as DNA repair and apoptosis. ROS may also alter genetic expression through a number of processes, such as protein adducts, DNA methylation, histone interactions, and through small noncoding RNAs [24]. Ionizing radiation interacts with DNA both directly and indirectly, by providing energy to the system and thereby releasing electrons from their atoms [24]. Free electrons may then interact with other atoms and molecules to form ROS that will lead to DNA damage inducing cellular damage and repair systems [32]. As well as the production of ROS through ionizing radiation exposures, GCs in sufficient quantity to overwhelm the placental barrier may increase levels of ROS and induce genetic changes, providing multiple potential processes for phenotypical changes [33,34].

Adverse behaviors, such as depressive and anxious tendencies, have been associated with late gestational prenatal stress. Critical periods of neural sensitivity to radiation lie between gestational days 11 and 17 in mice, where neurons are still growing and differentiating [8]. The hippocampal region is dense with GC receptors and is highly involved in learning and memory, including the reward and fear systems, by receiving input directly from the emotional processing center, the amygdala. In early gestation, significant hippocampal damage leads to memory and motor deficits in offspring exposed to radiation [35]. Different regions of the cortex are linked to risk aversion, social anxiety, and higher order cognition, whereas the cerebral cortex receives sensorimotor input and is responsible for perceptive processing. Early research showed structural damage to the cortices from x-irradiation on GD 15 [36]. The cerebellum has recently been noted to be more involved in cognition, addiction, and depression. Late gestational exposure at GD 21 to 2500 mGy x-irradiation and 1500 mGy cyclotron irradiation both resulted in significant changes in developing cerebella [37].

The current study examined the impact of low dose radiation during the late gestation on adult behavioural outcomes and environmental experiences (such as perception and learning), with a focus on relevant regions of the brain (cerebral cortex, hippocampus, and cerebellum). Gene analysis examined the effects of prenatal radiation exposure on DNA damage, ROS regulation, and synaptic activity to investigate different neural regions and potential genetic modifications.

## 2. Results

### 2.1. Behavioral Tests

Observational data for the Open Field Task measured exploratory activity as an indicator for anxiety (See Table 1). A main effect of sex on rearing activity indicated males (43.77 ± 1.55 rears) were more active than females (33.19 ± 1.94 rears) with Welch’s F (1, 57.969) = 18.217, *p* < 0.0001. No other behavioral measures were significant for this task and no effects of prenatal exposure to ionizing radiation were found. 

In the Social Interaction Task, animals were observed for anxiety in relation to a stranger mouse (See Table 2). There was a main effect of prenatal exposure to ionizing radiation on rearing behavior, λ = 0.659, F (12, 137.871) = 1.964, *p* = 0.032, η^2^ = 0.13. In contrast, the 1000 mGy offspring (41.25 ± 2.57 rears) were more active than Sham offspring (28.67 ± 2.78 rears). There were no other measurable behavioral differences in either the Social Interaction Task or the Porsolt Swim Task.

### 2.2. Genetic Analysis

**Figure 1:**
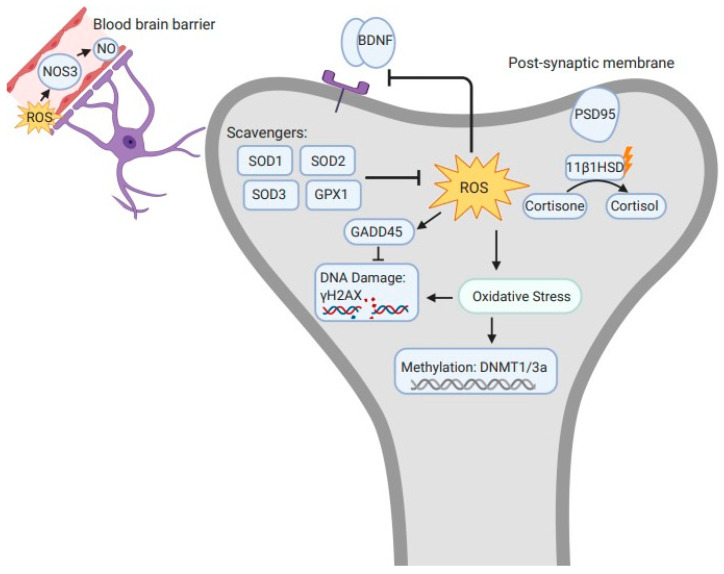
Related gene functions in relation to ROS after prenatal exposure to radiation. Dysregulated genes of C57Bl/6J brain regions. Each gene is differentially expressed depending upon region and animal sex. Endothelial nitric oxide (NOS3), superoxide dismutase 1, 2, 3 (SOD1, 2, 3), glutathione peroxidase 1 (GPX1), γH2AX, and post-synaptic density 95 (PSD95) were all upregulated. Growth arrest and DNA damage (GADD45), brain derived neurotrophic factor (BDNF), and DNA methyltransferase 1 and 3a (DNMT1/DNMT3a) were downregulated. Endothelial NOS leads to an increase in NO neurotransmission. ROS scavengers inhibit excess ROS. ROS inhibits BDNF and promotes oxidative stress, leading to DNA damage and methylation. Figure was created using biorender.com.

Few dysregulated genes (see Figure 1) were discovered per brain region for male and female adult mice exposed prenatally to sublethal doses of ionizing radiation. Out of 31 genes analyzed, across the treatment groups, 14 were dysregulated in the cerebella and the cortices, and 9 were dysregulated in the hippocampi (see Table 3, Table 4 and Table 5). In the cerebella, 7 genes were dysregulated in the 50 mGy condition, compared to 11 in the 300 mGy group, and 4 in the 1000 mGy. ROS regulator SOD1 was overexpressed relative to sham controls in the 50 mGy females with a fold change of 1.97 ± 0.34 (2^ΔΔCQ^ + S.E.M.; Table 3). The 50 mGy females also had a dysregulation in SOD2 (1.68 ± 0.15) and SOD3 (1.40 ± 0.16) genes. In the 300 mGy and 1000 mGy conditions, only SOD2 showed change with a decrease in activity in 300 mGy animals (0.46 ± 0.05) and an increase in 1000 mGy (1.11 ± 0.06) males. The p53 tumor suppressor was decreased in both males (0.58 ± 0.03) and females (0.57 ± 0.09). DNA methylation genes DNMT3a, DNMT1, and the circadian rhythm gene PER2 show similar dysregulation across treatment conditions. Synaptic activity genes such as synaptophysin, BDNF, PSD95, NeuN, and NeuroD have changes in activity in male and female offspring predominantly in the 300 mGy condition. Microglia and apoptotic genes TMEM119, CX3CR1, and BAX are all dysregulated in the 50 mGy and 300 mGy offspring.

In the cerebral cortices, the 50 mGy and 300 mGy conditions had 6 and 5 dysregulated genes respectively, whereas the 1000 mGy condition had 3. SOD1 was upregulated in 1000 mGy females (1.23 ± 0.08), whereas SOD2 was downregulated in both males (0.50 ± 0.19) and females (0.46 ± 0.06) in the 300 mGy condition. For SOD3, males in the 50 mGy condition were also downregulated (0.86 ± 0.04). Similar to the cerebella, the cortices also displayed dysregulation in DNA methylation, circadian rhythm, synaptic activity, microglia, and apoptotic genes; however, this was predominantly in the 50 mGy and 300 mGy conditions. In the hippocampi, the 50 mGy condition had 6 dysregulated genes compared to 4 and 3 in the 300 mGy and 1000 mGy conditions, respectively. Here, there were no expression changes in the DNA methylation genes, or microglia gene TMEM119. However, SOD1 males (1.47 ± 0.21) in the 50 mGy, males (1.70 ± 0.28) and females (1.52 ± 0.25) in the 300 mGy conditions displayed increased expression. SOD3 50 mGy females were decreased compared to sham controls (0.54 ± 0.15), similarly in p53 (0.40 ± 0.15). p53 in 300 mGy females was also decreased (0.81 ± 0.06). In the 1000 mGy condition, PER2 (0.88 ± 0.05), PSD95 (1.36 ± 0.22), and NeuN (0.72 ± 0.04) were the only significant genetic expression changes.

## 3. Discussion

During the late gestation developmental period, animals are susceptible to fetal programming under stressful or adverse in-utero conditions [17,18]. Late gestation is a period of differentiation, synaptogenesis, and neuronal migration; while not a critical period for survival, it is a sensitive period that requires a healthy environment for proper neural development [8]. Adverse neurological outcomes in both humans and rodents after prenatal exposure to various types of stress include decreased neurogenesis, impaired memory and learning, decreased synaptic plasticity, increased levels of depression, increased likelihood of attention deficit hyperactive disorder, and increased avoidance of novel situations [1,15,38,39,40]. The current study examined the impact of prenatal exposures to low doses of ionizing radiation on the behavioral and genetic profiles in specific brain regions of adult offspring. Our results did not exhibit any significant behavioral responses to the prenatal treatments; however, there are multiple genes dysregulated in the three brain regions across the dosage conditions.

C57Bl/6J mice are a common rodent model for a broad spectrum of research; however, previous literature has implicated they are resistant to radiation exposures [41,42,43]. In the present study, with doses of ionizing radiation of 1000 mGy and lower, we were unable to find adverse behavioral outcomes in correlation to prenatal exposures, aside from one main effect of the treatment on rearing behavior in the social interaction task at the 1000 mGy dose, an indication of hyperactivity. A main effect of sex on rearing in the open field task was also discovered—with males exhibiting higher activity than females—a departure from normal behavior for this breed of mouse on this task [44,45]. In isolation of other behavioral measures for each task, these are not indicative of any significant adverse behaviors. Specific to ionizing radiation, the period of organogenesis or embryonic development in C57Bl/6J mice has been shown to be neutrally sensitive to exposures less than 213 mGy, with cellular apoptosis and cognitive impairment as side effects [46,47]. Considering the critical period and the mid-gestational effects of ionizing radiation on C57Bl/6J animals, it is valuable to investigate late-gestation with this model; however, according to our lack of robust dose response and historical literature referring to C57Bl/6J mice as radiation-resistant, it is reasonable to expect that a behavioral response will only be measurable at higher doses of ionizing radiation [48].

There was a significant sex difference in gene dysregulation across the brain regions, which is a normal trend for brain region, stress type, and various mouse breeds [49,50]. The number of dysregulated genes in each pathway and brain region, however, is limited in the 1000 mGy condition but indicated a trend of oxidative stress and damage. In the cerebella, cerebral cortex, and hippocampus of the mice, three ROS regulatory genes, SOD1, SOD2, and SOD3 were dysregulated. SOD1, or superoxide dismutase 1, encodes for a cytoplasmic enzyme that will catalyze the dismutation of a free radical, such as superoxide, into hydrogen peroxide [51]. SOD genes work to reduce cellular stress due to ROS and act as an endogenous antioxidant system, indicating a level of stress-response in these regions were active (see Figure 1). Similarly, at 300 mGy, C57Bl/6j offspring have been shown to have SOD dysregulation in cardiac tissue [52].

Synaptic activity genes, post-synaptic density 95, synaptophysin, NeuroD, BDNF, NOS3, and NeuN were all differentially expressed in the different brain regions, especially in the 300 mGy cerebella. PSD95 is a synaptic anchoring protein in excitatory glutaminergic synapses, associated with synaptic plasticity. Disruption of the balance of excitatory versus inhibitory synapses, as indicated by the upregulation in female cerebral cortices, can increase AMPA receptors, changing synaptic strength by inhibiting long-term potentiation and enhancing long-term depression [53,54]. A link has been made between PSD95 overexpression and seizure activity, schizophrenia, and addictive behaviors [54]. Brain-derived neurotrophic factor or BDNF is involved in synaptic formation and neurogenesis. Mouse knock-out models of BDNF show severe synaptic impairments and downregulation of the gene is linked to major depressive disorder and chronic stress states [55,56,57,58].

Endothelial nitric oxide synthase (NOS3) can be dysregulated in the presence of ROS and promote nitric oxide neurotransmission and vascular changes [58]. PER2 is a circadian rhythm gene that, when disrupted, can lead to cognitive decline, impaired learning, and metabolic syndrome [59,60,61,62]. NeuroD has been noted to be a key gene in cellular differentiation during development within the cerebella and hippocampus [63,64]. NeuN is a biomarker for neurons; downregulation of this, along with PSD95, would indicate fewer cells in the region and a reduction of excitatory synaptic activity. In a study investigating the effects of PSD95 knockout mice, it was discovered that male and female mice exhibit oppositional behavioral phenotypes in sociability and activity; exhibited in the present study, it is shown that this is a genetic expression and behavioral difference [65].

## 4. Materials and Methods

### 4.1. Animals and Housing

All experimental protocols were approved by the Animal Care Committee of McMaster University (AUP-15-11-26) and are in accordance with the Canadian Council on Animal Care guidelines. The study was designed to have 8 males and 8 females per treatment condition, for a total of 4 treatment conditions. Male and female C57Bl/6J mice (8–12 weeks) were obtained from Jackson Laboratories (Bar Harbor, ME, USA) and bred at McMaster University (Hamilton, ON, Canada). Two females were placed in a cage overnight with a single male, for a total of 14 dams per treatment condition. Pregnancies were confirmed with the presence of a vaginal plug in the morning, which was considered gestational day (GD) 0. Pregnant females were then singly housed for the duration of the pregnancy. Normal rodent chow and water was provided ad-libitum (Teklad Diets Envigo, Madison, WI, USA); and animals were maintained on a 12:12 h (7 am–7 pm) light-dark cycle. Animals were housed in standard, non-ventilated cages.

### 4.2. Irradiation

Pregnant C57Bl/6 mice were exposed to ionizing radiation with a 662 keV, ^137^Cs γ-radiation Taylor Radiobiology source at McMaster University. Animals were transported in a temperature-controlled vehicle from the housing facility to the irradiation building in their home cages. Upon arrival, the cages were placed under shielding for acclimatization to the new location for 1 h prior to exposures. Cages were placed equidistant from the source and they received 10 mGy per minute dose on gestational day 15; fetal doses were measured at 8.9 mGy per minute [66]. Dam and fetal dose rates were obtained using transplanted thermoluminescent dosimeters as previously described [67]. Animals were randomly assigned to either sham, 50, 300, or 1000 mGy exposure conditions.

### 4.3. Offspring

A maximum of pups from each dam (n = 8 per sex) were utilized for behavioral and genetic testing. Pups were raised under normal conditions; 2–3 per cage and food and water provisioned *ad-libitum* until the age of 17–18 weeks. Pups then underwent behavioral testing and were euthanized by cervical dislocation within 24 h of the last task. Relevant tissues were harvested, frozen immediately with liquid nitrogen, and then stored at −80 °C until genetic analysis.

### 4.4. Behavioral Tests

All behavior was recorded utilizing video-cameras. Experimenters and observers were blind to treatment groups. Behavioral tasks were conducted in random order to prevent ordering effects and were completed over the course of 1.5 days [67,68]. Animals were brought into the testing rooms at least one hour prior to testing to acclimatize. All tasks were conducted during the light period and each task was washed between animal testing with 70% ethanol and water [69]. All behavior was scored using Behavioural Observation Research Initiative Software (BORIS) [70].

#### 4.4.1. Open Field Task

As a measure of general anxiety, four open field boxes (40 × 40 × 30 cm) were placed on the floor [71]. Mice were placed in the center of the task and were allowed to explore the novel environment for 5 min. Behavior measured by the observer included number of grid crossings to the center of the filed, total time spent within the center of the field, and the total number of rears. A grid was placed over the computer screen while utilizing BORIS software to create a central square measuring 20 × 20 cm. Grid crossings were defined as three or more paws across the line and rearing was defined as standing on two rear paws.

#### 4.4.2. Social Interaction Task

To measure social anxiety, animals were placed in a clean, normal housing cage for a total of 5 min with a stranger mouse [72]. The stranger mouse was matched for sex, but was not an experimental animal and was replaced with a new stranger after a few trials to prevent stress responses. The stranger was placed behind a plexiglass divider for protection and identification. The stranger mouse had a reduced area (3 inches wide) to explore in order to prevent it from hiding from interactions with the experimental animal. Observed behavior included rearing, as defined above, number of approaches, and total time in approach defined as being within 1.5 inches of the plexiglass, facing the stranger mouse.

#### 4.4.3. Porsolt Swim Task

Originally designed as a measure of despondency and learned helplessness, animals were placed in buckets of water, at 32 °C and were observed for a total of 5 min [73]. The water depth was maintained at 9 inches in 11 × 11-inch plastic buckets. Animals were placed in the center of the bucket and were dried off with a paper towel and placed back into their home cages for at least an hour prior to participating in any other behavioral task. Animals were monitored by observers nearby, but out-of-sight in case a rescue was required. No animals required rescuing. Observed behavior included latency-to-float and the total time spent immobile or floating. Immobility was defined as swimming cessation—movement involved in keeping the animal’s head above water or to push itself from bumping into the wall were included in the immobility measure.

### 4.5. Brain Dissections

Brain regions were determined utilizing Paxinos and Franklin’s mouse brain atlas [74]. The prefrontal cortex was delineated by a +2.2 mm anterior-posterior (AP) position from bregma. The cerebellum was removed using a scalpel, and the remaining cortex and hippocampus were removed using forceps and a scalpel [75]. All dissections were conducted in sterile petri dishes on top of ice.

### 4.6. Primer Design

Primers were designed using Primer Bank, Primer3, Primer3Plus, or Gemi; sequences and accession numbers are listed in Supplementary Table S1. The primer for BDNF was designed based on previous literature [76]. Primers were validated utilizing serial dilutions and specificity was analyzed using melt curves post amplification [77]. Genes were chosen relevant to pathways in ROS regulation, cortisol regulation, cell cycling, DNA damage and tumor suppression, DNA methylation, circadian rhythm, synaptic activity, apoptosis, and microglia activity.

### 4.7. RNA Extraction and Complimentary DNA Synthesis

Neural tissue was weighed and then mechanically homogenized with 1 mL of TRI Reagent (Sigma-Aldrich, St. Louis, MO, USA) per 50 mg of tissue in a TissueLyser (Qiagen, Hilden, Germany) for two 2-min cycles at 30 Hz. Supernatant was transferred to a fresh tube and 200 µL of chloroform per 1 mL of TRI reagent was added and vortexed [20,78,79]. After incubation at room temperature for 15 min, samples were then centrifuged at 12,000× *g* for 20 min at 4 °C. The top aqueous phase was removed and added into a fresh tube with 500 µL of isopropanol per 1 mL of TRI Reagent. Samples were then vortexed and incubated at room temperature for 10 min. After incubation, samples were again centrifuged, at 12,000× *g* for 8 min at 4 °C and pellets were washed with 1 mL of 70% ethanol per 1 mL of TRI Reagent. A final centrifugation at 7500× *g* for 5 min was conducted and RNA pellets were air dried prior to their resuspension in 20 µL of diethylpyrocarbonate (DEPC) treated nuclease-free water. Samples were then placed on the Thermomixer R (Eppendorf, Hamburg, Germany) for 10 min at 37 °C. Total RNA concentrations were measured using the spectrophotometric measurement of the absorbance at 260 nm (Nanodrop ND-1000, Nanodrop Technologies, Wilmington, DE, USA) [79]. RNA suspensions were stored at −80 °C for long term storage.

2 µg of total RNA was treated with DNAse I (Sigma-Aldrich) according to manufacturer guidelines prior to complimentary DNA (cDNA) synthesis. For cDNA synthesis, random primers (1 µg/1 µL, Roche Diagnostics, Basel, Switzerland) were added to the DNAse-treated RNA. Samples were vortexed, spun down, and then heated at 70 °C for 5 min in a thermal cycler (MJ Mini, Bio-Rad, Hercules, CA, USA) and then placed on ice. According to manufacturer’s guidelines, 2 U/µL Mu-MLV reverse transcriptase (Promega, 200 U/µL, Madison, WI, USA), mixed dNTPs (200 µM), M-MuLV 5× Reaction Buffer (Promega), and DEPC water were added to each sample. A negative control was prepared with no reverse transcriptase. Samples were mixed, spun down, and incubated for 60 min at 37 °C. The final concentration of cDNA samples was 0.04 µg/µL.

### 4.8. RT-qPCR

Utilizing the QuantStudio5 Real-Time PCR System (Applied Biosystems, Waltham, MA, USA), genes were analyzed comparing samples from the Sham, 50, 300, and 1000 mGy animals (n = 3 to 8). 12 µL reaction volumes were used with 0.3 ng/µL of cDNA of each sample, DEPC water, 2 ng/µL of forward and reverse primers, and SYBR green master-mix (SensiFAST SYBR Lo-ROX, Bioline, Saint Petersburg, Russia).

### 4.9. Statistical Analysis

All statistical analyses for behavioral and genetic comparisons were carried out using IBM SPSS 20.0. All conditions were an n = 8 per sex. Datasets were tested for normality and homogeneity of variance using Shapiro-Wilk and Levene’s test, *p* > 0.05. Inter-rater reliability was calculated using Pearson’s Correlation, r = 0.993. General linear model analysis of variance (ANOVA) was conducted, otherwise Welch’s test was utilized with alpha levels set to *p* = 0.05. All results are presented in mean ± standard error of the mean (S.E.M). Post-hoc analyses were conducted where appropriate, using Tukey’s Honestly Significant Difference (HSD).

## 5. Conclusions

According to our behavioral results, the pattern of genetic disruption in both male and female offspring lead us to believe that the threshold for fetal programming of adverse coping behavior and anxious behavior is just beyond the doses we have utilized here. These animals are displaying a neural response to prenatal stress exposures that either do not elicit behavioral changes at this level of disruption, or display changes that we have not investigated, such as late-cognitive decline or schizophrenic-behavior as associated with the gene dysregulation patterns in other studies [44,48]. Although changes in certain genes examined were observed with prenatal exposure to ionizing radiation (50 mGy, 300 mGy, and 1000 mGy), these changes in gene expression had no correlation or impact on the behavioral outcome in the adult offspring.

## 6. Limitations

The authors recognize that different behavioral tasks targeting other cognitive processes may show significant changes in behavioral phenotypes that are not present in the tasks we have chosen. We also acknowledge that future work should include larger sample sizes to ensure confidence.

## Figures and Tables

**Table 1:** Observational Data on Open Field Task.

Behavior	Sex	Sham	50 mGy	300 mGy	1000 mGy	Results
Rears	M F	44.83 ± 3.74 34.75 ± 3.24	39.67 ± 3.06 29.25 ± 3.24	50.00 ± 3.24 40.63 ± 3.24	41.00 ± 3.47 28.86 ± 3.47	Sex effect: *p* < 0.0001
Grid Crossings	M F	52.83 ± 6.07 65.38 ± 5.26	52.56 ± 4.96 53.63 ± 5.26	55.38 ± 5.26 55.00 ± 5.26	57.71 ± 5.62 62.29 ± 5.62	Not significant
Time in Center	M F	184.14 ± 14.1 181.92 ± 12.2	197.29 ± 11.5 189.37 ± 12.2	199.48 ± 12.2 183.84 ± 12.2	194.80 ± 13.01 175.23 ± 12.2	Not significant

The mean and S.E.M of each measured behavior represented as occurrences of the behavior, except for Time in Center, which is measured in seconds. For rearing behavior, males were significantly more active than females on average.

**Table 2:** Observational Data on Social Interaction Task.

Behavior	Sex	Sham	50 mGy	300 mGy	1000 mGy	Results
Rears	M F	26.33 ± 4.20 31.00 ± 3.63	35.44 ± 3.43 38.75 ± 3.63	33.25 ± 3.63 39.50 ± 3.63	44.38 ± 3.63 38.13 ± 3.63	Dose effect: *p* = 0.01
# of Approaches	M F	28.50 ± 2.35 23.75 ± 2.04	25.56 ± 1.92 25.50 ± 2.04	23.00 ± 2.03 28.13 ± 2.04	30.50 ± 2.04 27.50 ± 2.04	Not significant
Total Time in Approach	M F	152.83 ± 17.09 121.25 ± 14.80	141.00 ± 13.95 145.38 ± 14.80	121.89 ± 14.80 139.25 ± 14.80	162.00 ± 14.80 111.63 ± 14.80	Not significant

The mean and S.E.M of each measured behavior represented as occurrences, except for Total Time in Approach, which is measured in seconds. 1000 mGy animals were significantly more active in rearing than shams.

**Table 3:** Relative Gene Expression in the Cerebella of 50, 300, and 1000 mGy Offspring.

Gene Expression in 50, 300, and 1000 mGy Offspring Cerebella Relative to Sham (Fold Change ± SEM)				
**ROS Regulation**				
**Gene**	**Sex**	**50 mGy**	**300 mGy**	**1000 mGy**
SOD1	M F	1.46 ± 0.47 **1.97 ± 0.34**	nd	1.00 ± 0.18 0.97 ± 0.13
SOD2	M F	1.02 ± 0.29 **1.68 ± 0.15**	**0.46 ± 0.05** 0.74 ± 0.17	**1.11 ± 0.06** 1.01 ± 0.08
SOD3	M F	1.12 ± 0.16 **1.40 ± 0.16**	0.85 ± 0.07 1.16 ± 0.12	1.03 ± 0.55 0.94 ± 0.31
**DNA Damage & Tumor Suppression**				
p53	M F	0.74 ± 0.25 1.04 ± 0.02	**0.58 ± 0.03** **0.57 ± 0.09**	1.05 ± 0.13 0.87 ± 0.06
**DNA Methylation**				
DNMT3a	M F	**1.66 ± 0.24** 1.20 ± 0.14	1.44 ± 0.29 1.02 ± 0.09	1.24 ± 0.17 **0.81 ± 0.06**
DNMT1	M F	0.49 ± 0.31 0.81 ± 0.59	0.80 ± 0.11 **0.69 ± 0.07**	1.07 ± 0.14 **0.71 ± 0.07**
**Circadian Rhythm**				
PER2	M F	1.80 ± 0.59 1.59 ± 0.34	0.67 ± 0.19 **0.47 ± 0.15**	0.82 ± 0.19 0.79 ± 0.14
**Synaptic Activity**				
Synaptophysin	M F	0.54 ± 0.18 1.16 ± 0.13	**0.65 ± 0.04** **0.46 ± 0.11**	*1.09 ± 0.07* *0.94 ± 0.04*
BDNF	M F	1.34 ± 0.20 **1.64 ± 0.12**	1.16 ± 0.07 **1.33 ± 0.17**	1.14 ± 0.16 1.06 ± 0.20
NOS3	M F	1.83 ± 0.42 1.23 ± 0.12	**0.46 ± 0.11** **0.54 ± 0.09**	nd
PSD95	M F	nd	1.31 ± 0.20 2.64 ± 1.22	**1.55 ± 0.32** 0.79 ± 0.13
NeuN	M F	0.54 ± 0.21 1.09 ± 0.16	0.85 ± 0.09 0.81 ± 0.11	0.74 ± 0.29 0.73 ± 0.24
NeuroD	M F	1.00 ± 0.88 0.94 ± 0.72	**2.22 ± 0.80** **2.47 ± 0.72**	0.82 ± 0.35 0.75 ± 0.24
**Microglia**				
TMEM119	M F	**2.06 ± 0.62** 1.56 ± 0.56	**0.37 ± 0.06** **0.32 ± 0.10**	1.13 ± 0.47 0.86 ± 0.36
**Apoptosis**				
CX3CR1	M F	1.41 ± 0.37 0.90 ± 0.09	**0.33 ± 0.04** **0.47 ± 0.13**	0.90 ± 0.59 0.93 ± 0.45
BAX	M F	0.77 ± 0.14 **1.51 ± 0.31**	**1.20 ± 0.06** 1.06 ± 0.10	0.80 ± 0.28 1.23 ± 0.47
APAF1	M F	nd	nd	0.82 ± 0.21 0.76 ± 0.23

Fold change of 50, 300, and 1000 mGy males and females compared to Sham controls (n = 3 to 8). Bolded numbers are significant compared to controls. Sex differences are in italics. Genes not detected due to multiple melt curve peaks were marked with nd. All significances are *p* ≤ 0.05.

**Table 4:** Relative Gene Expression in the Cortices of 50, 300, and 1000 mGy Offspring.

Gene Expression in 50, 300, and 1000 mGy Offspring Cortices Relative to Sham (Fold Change ± SEM)				
**ROS Regulation**				
**Gene**	**Sex**	**50 mGy**	**300 mGy**	**1000 mGy**
SOD1	M F	1.86 ± 0.73 1.43 ± 2.21	nd	1.08 ± 0.14 **1.23 ± 0.08**
SOD2	M F	1.40 ± 0.41 0.72 ± 0.24	**0.50 ± 0.19** **0.46 ± 0.06**	1.12 ± 0.14 1.04 ± 0.04
SOD3	M F	**0.86 ± 0.04** 1.83 ± 0.85	0.95 ± 0.05 0.88 ± 0.06	1.08 ± 0.13 0.97 ± 0.20
**DNA Damage & Tumor Suppression**				
p53	M F	0.48 ± 0.20 **0.70 ± 0.08**	0.74 ± 0.11 **0.54 ± 0.12**	0.97 ± 0.19 1.04 ± 0.01
**DNA Methylation**				
DNMT3a	M F	0.98 ± 0.06 1.93 ± 0.91	2.09 ± 1.03 0.99 ± 0.11	1.01 ± 0.08 0.76 ± 0.11
DNMT1	M F	0.84 ± 0.57 0.56 ± 0.18	**0.65 ± 0.13** **0.56 ± 0.13**	0.90 ± 0.09 1.00 ± 0.06
**Circadian Rhythm**				
PER2	M F	0.99 ± 0.16 0.74 ± 0.13	**0.66 ± 0.06** **0.33 ± 0.08**	0.80 ± 0.24 0.85 ± 0.14
**Synaptic Activity**				
Synaptophysin	M F	0.87 ± 0.61 0.40 ± 0.21	0.63 ± 0.19 0.71 ± 0.19	1.16 ± 0.18 0.76 ± 0.13
BDNF	M F	0.85 ± 0.43 1.64 ± 0.12	1.15 ± 0.36 0.91 ± 0.19	**0.57 ± 0.06** 0.86 ± 0.30
NOS3	M F	nd	**0.42 ± 0.06** **0.52 ± 0.16**	1.03 ± 0.31 1.40 ± 0.27
PSD95	M F	nd	1.12 ± 0.24 0.77 ± 0.17	1.00 ± 0.32 **1.99 ± 0.39**
NeuN	M F	0.54 ± 0.19 0.37 ± 1.48	0.64 ± 0.11 0.68 ± 0.17	0.83 ± 0.15 1.46 ± 0.29
NeuroD	M F	**0.39 ± 0.20** 1.50 ± 1.83	1.16 ± 0.32 1.18 ± 0.37	1.00 ± 0.17 1.13 ± 0.30
**Microglia**				
TMEM119	M F	**1.77 ± 0.44** 1.59 ± 1.32	nd	1.13 ± 0.47 0.86 ± 0.36
**Apoptosis**				
CX3CR1	M F	0.52 ± 0.19 0.85 ± 0.07	**0.44 ± 0.08** **0.35 ± 0.05**	1.28 ± 0.13 1.26 ± 0.23
BAX	M F	**0.73 ± 0.07** 0.85 ± 0.09	0.88 ± 0.16 1.11 ± 0.19	0.89 ± 0.21 1.36 ± 0.37
APAF1	M F	0.88 ± 0.12 0.78 ± 0.35	nd	1.49 ± 0.28 1.29 ± 0.39

Fold change of 50, 300, and 1000 mGy males and females compared to Sham controls (n = 3 to 8). Bolded numbers are significant compared to controls. Sex differences are in italics. Genes not detected due to multiple melt curve peaks were marked with nd. All significances are *p* ≤ 0.05.

**Table 5:** Relative Gene Expression in the Hippocampi of 50, 300, and 1000 mGy Offspring.

Gene Expression in 50, 300, and 1000 mGy Offspring Hippocampi Relative to Sham (Fold Change ± SEM)				
**ROS Regulation**				
**Gene**	**Sex**	**50 mGy**	**300 mGy**	**1000 mGy**
SOD1	M F	**1.47 ± 0.21** 0.70 ± 0.23	**1.70 ± 0.28** **1.52 ± 0.25**	1.09 ± 0.27 1.14 ± 0.09
SOD2	M F	1.27 ± 0.21 0.78 ± 0.24	1.21 ± 0.42 1.08 ± 0.58	1.29 ± 0.21 0.94 ± 0.07
SOD3	M F	0.90 ± 0.09 **0.54 ± 0.15**	1.10 ± 0.20 1.10 ± 0.15	*1.11 ± 0.27* *1.28 ± 0.27*
**DNA Damage & Tumor Suppression**				
p53	M F	1.05 ± 0.07 **0.40 ± 0.15**	1.19 ± 0.18 **0.81 ± 0.06**	1.83 ± 1.55 0.89 ± 0.63
**DNA Methylation**				
DNMT3a	M F	0.92 ± 0.12 0.54 ± 0.15	1.01 ± 0.20 0.89 ± 0.09	1.07 ± 0.22 0.97 ± 0.05
DNMT1	M F	1.07 ± 0.12 0.74 ± 0.22	1.04 ± 0.18 1.04 ± 0.11	1.17 ± 0.20 0.96 ± 0.07
**Circadian Rhythm**				
PER2	M F	0.82 ± 0.08 0.84 ± 0.26	0.87 ± 0.14 0.99 ± 0.10	1.01 ± 0.23 **0.88 ± 0.05**
**Synaptic Activity**				
Synaptophysin	M F	0.85 ± 0.09 **0.53 ± 0.15**	1.08 ± 0.18 0.84 ± 0.16	1.26 ± 0.14 0.82 ± 0.11
BDNF	M F	0.70 ± 0.18 **0.57 ± 0.16**	1.62 ± 0.51 1.15 ± 0.18	1.19 ± 0.33 0.74 ± 0.21
NOS3	M F	0.79 ± 0.12 1.02 ± 0.47	nd	nd
PSD95	M F	nd	0.65 ± 0.31 0.81 ± 0.16	1.80 ± 0.54 **1.36 ± 0.22**
NeuN	M F	1.05 ± 0.15 0.75 ± 0.20	0.83 ± 0.13 0.87 ± 0.13	1.04 ± 0.33 **0.72 ± 0.04**
NeuroD	M F	1.13 ± 0.27 0.98 ± 0.19	1.79 ± 0.72 **1.80 ± 0.38**	1.80 ± 0.75 0.76 ± 0.28
**Microglia**				
TMEM119	M F	1.07 ± 0.12 0.75 ± 0.36	1.03 ± 0.15 1.08 ± 0.11	1.18 ± 0.17 1.13 ± 0.26
**Apoptosis**				
CX3CR1	M F	1.11 ± 0.11 0.68 ± 0.30	1.04 ± 0.09 0.97 ± 0.10	1.21 ± 0.17 1.11 ± 0.08
BAX	M F	0.86 ± 0.07 **0.68 ± 0.05**	**1.31 ± 0.08** 1.22 ± 0.13	1.02 ± 0.28 0.82 ± 0.14
APAF1	M F	nd	nd	1.20 ± 0.17 0.99 ± 0.14

Fold change of 50, 300, and 1000 mGy males and females compared to Sham controls (n = 3 to 8). Bolded numbers are significant compared to controls. Sex differences are in italics. Genes not detected due to multiple melt curve peaks were marked with nd. All significances are *p* ≤ 0.05.

## Data Availability

Datasets will be made available upon request.

## Supplementary Materials

Supplementary materials can be found at https://www.mdpi.com/article/10.3390/ijms24108466/s1.
