# Supplementary material for: Absence of Depressive and Anxious Behavior with Genetic Dysregulation in Adult C57Bl/6J Mice after Prenatal Exposure to Ionizing Radiation
Source: Int J Mol Sci. 2023 May 9;24(10):8466. doi: 10.3390/ijms24108466 (PMC10217960; doi:10.3390/ijms24108466)
Supplement: Supplementary file 1 [file ijms-24-08466-s001.zip › ijms-2354351-Supplementary Materials.pdf]

Supplementary Information

Table S1. Primer Design

Pathway	Gene	Accession #	Forward Primer (5’ → 3’)	Reverse Primer (5’ → 3’)
ROS Regulation	SOD1	NM_011434	CGGATGAAGAGAGGCATGTTGGA	CAATGATGGAATGCTCTCCTGAG
	SOD2	NM_013671	TTACGACTATGGCGCGCTGGA	TCGTGGTACTTCTCCTCGGTG
	SOD3	NM_011435	GGCCTGTGGCTCTGTCACC	CCTATCTTCTCAACCAGGTCAAG
	Catalase	NM_009804	CCTGACATGGTCTGGGAC	CCATAGCCATTCATGTGCCG
	GPX1	NM_008160	GGTGCTGCTCATTGAGAATGTCG	GGGAAACCGAGCACCACCAG
Cortisol Regulation	HSD11β1	NM_001044751	GAAGAGTTCAGACCAGAAATGCT	CAATACCACATGGGCTCCCATTT
	HSD11β2	NM_008289	TCTTTGGTGCACTTGAGCTGACC	AGGCTGCCAAGCAGGGGTATG
	GR	NM_001361209	AGGCCGCTCAGTGTTTTCTA	TACAGCTTCCACACGTCAGC
Cell Cycle	BTG2	NM_007393.3	GGCTGTATTCCCCTCCATCG	CCAGTTGGTAACAATGCCATGT
DNA Damage & Tumor Suppression	γH2AX	NM_010436	GCCTCTCAGGAGTACTGAGGG	CCCGAAGTGGCTCAGCTCTTT
	p53	AB020317	GGAAGACTCCAGTGGGAACC	TCTTCTGTACGGCGGTCTCT
	GADD45A	NM_007836	GAAAGGATGGACACGGTGGG	GGGTCTACGTTGAGCAGC
	DDB2	NM_028119.5	ACCGAGTACGTCATGGCTCCC	CTTGGCTTCGGGCTCCAGCT
	MDM2	NM_001288586	CCTGGATCAGGATTCAGTTTCTG	TCATCATCCTCATCTGAGAGCTC
DNA Methylation	DNMT3A	NM_007872	GAGGGAACTGAGACCCCAC	CTGGAAGGTGAGTCTTGGCA
	DNMT3B	NM_001122997	AGCGGGTATGAGGAGTGCAT	GGGAGCATCCTTCGTGTCTG
	DNMT1	NM_001199433	AAGCCTGGTGTTGTCTACCGAC	CATCCAGGTTGCTCCCCTTG
Circadian Rhythm	Per2	NM_011066	ACAAGAAGGCCAAGGGGAAGG	GGCTCTCACTGGACATTAGCAG
Synaptic Activity	Doublecortin	NM_001110222	ATGTCAACCGGGAAAGCACA	TGGTGGAACCACAGCAACTT
	NeuN	NM_001039168	GGCAAATGTTCGGGCAATTCG	TCAATTTTCCGTCCCTCTACGAT
	NeuroD	NM_010894.2	ATGACCAAATCATACAGCGAGAG	TCTGCCTCGTGTTCCTCGT
	PSD-95	NM_007864.3	GGCGGAGAGGAACTTGTCC	AGAATTGGCCTTGAGGGAGGA
	Synaptophysin	NM_009305.2	CAGTTCCGGGTGGTCAAGG	ACTCTCCGTCTTGTTGGCAC
	NOS3	NM_008713	TTTGCTGCCCTTGGCCTGCG	CTCTGAACTCATGTACCAGCCG
	NRF2	NM_010902	CAGCACATCCAGACAGACACCA	TGGGAATGTCTCTGCCAAAAGCT
Microglia	TMEM119	NM_146162	TTCACCCAGAGCTGGTTCCATA	GAGTGACACAGAGTAGGCCA
Apoptosis	CX3CR1	NM_009987.4	TCACCGTCATCAGCATCGAC	CGCCCAGACTAATGGTGACA
	BAX	NM_007527	CTGGATCCAAGACCAGGGTG	GTGAGGACTCCAGCCACAAA
	BCL2	NM_009741	GCGTCAACAGGGAGATGTCA	GCATGCTGGGGCCATATAGT
	APAF1	NM_001282947.1	CGTCTTCCAGTGTAAGGACAGT	CCATAGATGGTGACCCACCC
Reference Genes	Beta Actin	NM_007393.3	GGCTGTATTCCCCTCCATCG	CCAGTTGGTAACAATGCCATGT

	RPL29	NM_009082.2	ACATGGCCAAGTCCAAGAAC	TGCATCTTCTTCAGGCCTTT

All primers listed were designed using Primer Bank, Primer3, Primer3Plus, or Gemi. Annealing temperature was 58 degrees Celsius.
